# Factors associated with medication adherence among children with rheumatic diseases

**DOI:** 10.3389/fphar.2023.1149320

**Published:** 2023-05-05

**Authors:** Roongroj Manatpreeprem, Butsabong Lerkvaleekul, Soamarat Vilaiyuk

**Affiliations:** Rheumatology Division, Pediatric Department, Faculty of Medicine Ramathibodi Hospital, Mahidol University, Bangkok, Thailand

**Keywords:** connective tissue disease, juvenile idiopathic arthritis, poor compliance, enthesitis-related arthritis, polyarticular juvenile idiopathic arthritis, pediatric rheumatology adherence questionnaire, PRAQ, pill count method

## Abstract

**Introduction:** Failure to take medications regularly leads to poorer health outcomes. The Pediatric Rheumatology Adherence Questionnaire (PRAQ) is an effective tool for assessing medication adherence in rheumatic patients. Therefore, we aimed to determine the factors associated with poor medication adherence among children with rheumatic diseases.

**Methods:** This was a cross-sectional study. Patients with rheumatic diseases who had taken at least one medication and had been followed up at our pediatric rheumatology clinic were included in the study, together with their caregivers. Patients with poor medication adherence were characterized as those who had taken less than 80% of their prescribed drugs, as determined using the pill count method. The original PRAQ was translated and validated in Thai language and was completed by caregivers and literate patients over age 13 years. Interviewing for additional problems with taking medications was conducted. We performed descriptive and logistic regression analyses.

**Results:** From 210 patients, 52.86% had juvenile idiopathic arthritis (JIA), and 46.19% had connective tissue diseases. The mean patient age was 14.10 ± 4.74 years, with a median (interquartile range) disease duration of 4.33 (2.08–6.98) years. PRAQ scores in the group with poor adherence were significantly higher than scores in the group with good adherence (11.00 ± 3.47 vs. 9.51 ± 3.16, *p* = 0.004). Enthesitis-related arthritis (ERA) (odds ratio [OR] 9.09, 95% confidence interval [CI] 1.25–66.18; *p* = 0.029) and polyarticular JIA (OR 6.43, 95% CI 1.30–31.75; *p* = 0.022) were associated with poor treatment adherence. Disease duration ≥5 years (OR 3.88, 95% CI 1.17–12.87; *p* = 0.027), active disease (OR 6.49, 95% CI 1.76–23.99; *p* = 0.005), PRAQ scores ≥12 (OR 6.48, 95% CI 1.76–23.82; *p* = 0.005), forgetting to take medications (OR 14.18, 95% CI 4.21–47.73; *p* < 0.001), and unawareness about the importance of the medicines (OR 44.18, 95% CI 11.30–172.73; *p* < 0.001) were predictors of poor drug adherence.

**Conclusion:** In the present study, poor medication adherence was found in one-fourth of children with rheumatic illnesses, particularly those with ERA, polyarticular JIA, longer disease duration, active disease, and high PRAQ scores. The most frequent reasons for inadequate medication adherence were forgetfulness and unawareness about the importance of disease control and consistency with treatment.

## 1 Introduction

Once patients develop refractory diseases, there are several aspects to consider, such as progressiveness of the disease, the efficacy of drugs, poor absorption of medicines, or incorrect administration techniques. An essential issue that is widely observed is poor drug adherence, particularly among adolescent patients with chronic illness. The term “non-adherence to medication” is defined as inconsistent behavior with respect to a pharmacological and non-pharmacological treatment prescribed by a healthcare provider (Sabaté, 2003). Rheumatic diseases are a type of chronic illness in children that require ongoing long-term monitoring and treatment (Favier, et al., 2020). Although medical treatment for rheumatic diseases is now very advanced, the common impediment of poor adherence has persisted for decades globally and has increased over time. The World Health Organization (WHO) has recognized this issue and released a report entitled “Adherence to Long-Term Therapies: Evidence for Action” for policymakers and healthcare administrators, who have an impact on national or local policies to improve adherence rates, leading to better long-term outcomes of chronic illness (Sabaté, 2003). One suggestion is that medication adherence is not only the responsibility of patients but also requires the support of healthcare providers and caregivers.

The frequency of poor medication adherence in rheumatic illnesses is varied (Feldman et al., 2007; Lawson et al., 2011; Bugni et al., 2012; Senzaki, 2015; Adriano et al., 2017; Keppeke et al., 2018; Nitinantaponk and Oo-puthinan, 2018; Scalzi et al., 2018), and poor medication adherence affects disease outcomes and healthcare costs (Feldman et al., 2007; McGrady and Hommel, 2013; Len et al., 2014; Pasma et al., 2017). The WHO has grouped factors associated with medication adherence into five dimensions: 1) social and economic factors, 2) healthcare team and system-related factors, 3) condition-related factors, 4) therapy-related factors, and 5) patient-related factors. Multiple barriers can affect a patient’s ability to adhere to their treatment; therefore, enhancing patients’ compliance requires comprehensive evaluation of all factors (Sabaté, 2003).

There are many ways to assess compliance in patients with a rheumatic disease, including quantitative (e.g., drug level, pill count) and qualitative (e.g., questionnaire, interviewing) methods. However, some measures have not been validated in children (Hansen et al., 2009; Lam and Fresco, 2015; Driscoll and Modi, 2020; Favier et al., 2020). Self-report and questionnaire-based methods are widely applied in rheumatic diseases because they are practical and yield more satisfying results than quantitative methods. Moreover, a questionnaire can also be used to assess the reasons for medication non-adherence. In a pilot study, the Pediatric Rheumatology Adherence Questionnaire (PRAQ), developed specifically for children with rheumatic diseases, showed a negative correlation with poor medication adherence in the socioeconomic index (Silva et al., 2019). This study selected quantitative and qualitative methods to comprehensively assess patients, including the pill count method, PRAQ questionnaire, and interviews. We aimed to determine the medication adherence rate and variables associated with poor medication adherence in children with rheumatic diseases to improve early recognition of these factors and thereby improve medication adherence rates and long-term outcomes of rheumatic diseases in children.

## 2 Materials and methods

This study was approved by the Research and Ethics Committee of the Faculty of Medicine Ramathibodi Hospital, Mahidol University (ethic number MURA 2021/358), and was carried out in accordance with the Declaration of Helsinki. Written informed consent was obtained from parents and patients aged more than 13 years prior to the study enrollment. In this study, we used a cross-sectional design. We included caregivers and patients taking at least one prescription drug who attended the pediatric rheumatology clinic of the Faculty of Medicine, Ramathibodi Hospital in Bangkok, Thailand between May 2021 and January 2022. Patients were diagnosed with juvenile idiopathic arthritis (JIA), systemic lupus erythematosus (SLE), and juvenile dermatomyositis (JDM), according to the International League Associations of Rheumatology classification of JIA (Petty et al., 2004), the 2019 European League Against Rheumatism and the American College of Rheumatology classification criteria for SLE (Aringer et al., 2019), and Bohan and Peter diagnosis criteria for definite diagnosis of JDM (Bohan and Peter, 1975a; Bohan and Peter, 1975b), respectively.

### 2.1 Data collection

Demographic and clinical data were collected, including age, sex, educational level, diagnosis, onset and duration of disease, disease status, total income, expenses for each visit, and primary caregiver. Disease status was classified into inactive or active disease for each disease. Inactive disease status for particular diseases was classified according to their specific criteria, which comprised Wallace’s criteria for inactive disease in JIA (Wallace et al., 2011) and Pediatric Rheumatology International Trials Organization criteria for clinically inactive disease in JDM (Lazarevic et al., 2013). For SLE, we used the clinical SLE disease activity index 2000 (cSLEDAI-2K) (Uribe et al., 2004) to measure disease activity. Inactive disease was defined as cSLEDAI-2K equal to zero (van Vollenhoven et al., 2017; Lerkvaleekul et al., 2022). All those apart from those mentioned above were categorized as active disease.

Information regarding all received medications was collected, including non-steroidal anti-inflammatory drugs (NSAIDs), disease-modifying antirheumatic drugs (DMARDs), biologic agents, and prednisolone. The patient or a caregiver who was the patient’s primary care provider was interviewed about the causes of irregular drug use and asked to self-evaluate the adherence to medication between visits. If caregivers believed that the child took 80% or more of prescribed medications between visits, this was considered “good adherence.” This definition was used for self-evaluation by patients as well.

### 2.2 PRAQ questionnaire

The PRAQ, developed by Silva et al. (2019), is used to assess adherence to treatment in pediatric chronic rheumatic diseases. The scale consisted of 25 dichotomous questions categorized into five indexes: 1) socioeconomic index, 2) healthcare team and system index, 3) health condition index, 4) therapeutic index, and 5) patient/caregiver index. Following permission granted by the creator of the PRAQ, two independent translators translated and cross-culturally adapted this questionnaire into Thai language. The Thai PRAQ version was summarized by two expert pediatric rheumatologists (SV, BL) and translators. Two independent translators performed back translation into English. The preliminary finalized version of the Thai PRAQ was evaluated in a pilot study of 20 caregivers. Questions with less than 80% agreement for comprehensibility were revised. The final version was reviewed and approved by the expert committee. The Thai children’s PRAQ was developed by modifying the original PRAQ questionnaire. We changed the wording to simplified language for children to comprehend. We removed items 3, 4, 5, 10, and 20, as these questions were only related to the caregiver’s context and did not apply directly to patients (Supplementary Table S1). The Thai children’s PRAQ was tested in a pilot sample of 20 literate patients aged more than 13 years. Questions with less than 80% agreement of comprehensibility were revised. We counted one point for answers of “Yes.” Total scores on the Thai version of the PRAQ for caregivers and the Thai PRAQ for patients were 25 and 20, respectively. Higher scores indicate greater risk of poor medication adherence in the patient.

### 2.3 Medication adherence

We used the pill count method to estimate drug adherence, calculated as the percentage of pills taken (dispensed drugs minus the remaining drugs at each visit) divided by the expected amount of drugs used (number of pills per day multiplied by the number of days between visits). Patients were defined as having good adherence if they had taken 80% or more of their prescribed medication (Hansen et al., 2009; Pasma et al., 2017). For patients taking more than one medication, we counted each drug separately. As in the previous definition, we categorized patients into the poor adherence group if at least one medication was taken irregularly.

### 2.4 Statistical analysis

Categorical variables are presented as percentage, and continuous variables are described with mean and standard deviation or median and interquartile range (IQR), as appropriate. Comparisons between groups were performed using an independent samples *t*-test, chi-squared test, or Fisher’s exact test. Logistic regression analysis was used to analyze the association with medication adherence. For the PRAQ, internal consistency was analyzed using Cronbach’s alpha coefficient with values < 0.5, 0.5–0.59, 0.6–0.69, 0.7–0.79, 0.8–0.89, and 0.9–1.0 considered unacceptable, poor, questionable, acceptable, good, and excellent internal consistency, respectively. Test–retest reliability was analyzed using the intraclass correlation coefficient (ICC) with values < 0.4, 0.4–0.59, 0.6–0.74, and 0.75–1.0 considered poor, fair, good, and excellent reliability, respectively. Statistical analysis was performed using IBM SPSS version 21 (IBM Corp., Armonk, NY, United States), and statistical significance was set at a *p*-value of <0.05.

## 3 Results

Among a total of 210 patients with rheumatic disease, 68.10% were girls, with a mean age of 14.10 ± 4.74 years. Approximately half of patients were diagnosed with JIA, among which polyarticular JIA (29.73%) and enthesitis-related arthritis (ERA) (26.13%) were the two most common JIA subtypes in our study population; these were followed by 97 (46.19%) patients with connective tissue diseases (CTDs), including SLE (79.38%) and JDM (16.49%). The median (IQR) disease duration was 4.33 (2.08–6.98) years, and 78 (37.14%) patients had active disease. Around half of patients were given their medicines by their caregiver, and most caregivers were the patient’s mother (74.29%), followed by the patient’s father (14.76%) and a grandparent or other relative (10.95%). The mean age of caregivers was 45.41 ± 8.36 years. The median number and form of medication was three tablets, and approximately one-fifth of patients received subcutaneous injections. Half of the patients were in secondary school, and most caregivers had a bachelor’s degree or higher. Regarding health insurance, approximately two-thirds of patients were enrolled in the universal Coverage Scheme (UCS), 17.62% were registered in the Civil Servant Medical Benefit Scheme, and 15.71% had no health insurance coverage.

### 3.1 PRAQ performance

In the pilot sample, comprehensibility of the preliminary version of the Thai PRAQ was 100% agreement for all questions. The expert committee assessed and approved the final version with 100% agreement. Twenty caregivers completed the PRAQ test–retest at a 4-week interval. The Thai PRAQ revealed acceptable internal consistency (Cronbach’s alpha 0.703) and excellent reliability (ICC 0.75–1.0). The data for the original PRAQ, the Thai caregiver version, and the Thai patient version are shown in Supplementary Table S1.

### 3.2 Medication adherence

**FIGURE 1:**
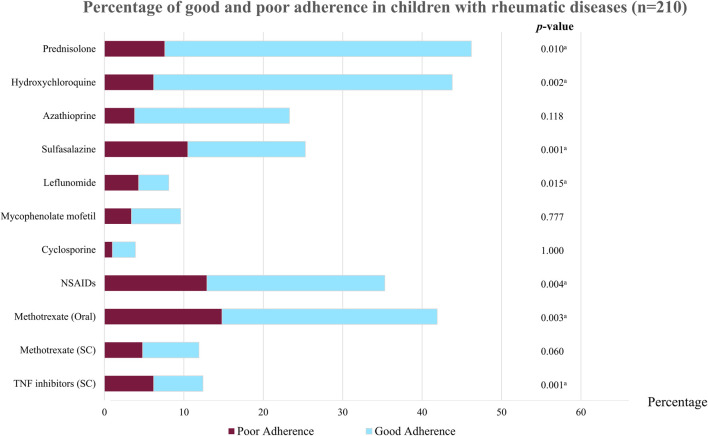
Percentage of good and poor adherence in children with rheumatic diseases (n = 210). NSAIDs, non-steroidal anti-inflammatory drugs; TNF, tumor necrosis factor; SC, subcutaneous. ^a^p < 0.05 indicates statistical significance.

A total of 158 (75.24%) patients had good adherence to medical treatment and 52 (24.76%) had poor adherence, as defined using the pill count method. A comparison of baseline characteristics between good and poor adherence groups is shown in Table 1; Figure 1. Most patients in the good adherence group were younger girls at study enrollment. A higher number of patients with CTDs had good adherence than patients with JIA. More patients with JIA subtypes, especially ERA (*p* < 0.001) and polyarticular JIA (*p* = 0.003), had poor adherence than good adherence. Patients with poor adherence had longer disease duration than those with good adherence (median 5.67, IQR 3.31–9.21 vs. 3.91, IQR 1.90–6.52; *p* = 0.005). Additionally, patients with active disease were prone to having inadequate medication adherence. The maximum number of tablets taken per day was higher in the poor adherence group than in the good adherence group (*p* < 0.001). Among patients in the poor adherence group, the medications most often taken irregularly were sulfasalazine, leflunomide, and mycophenolate mofetil (Supplementary Table S2). However, when patients or caregivers in the non-adherence group were asked to self-evaluate their medication use, approximately half estimated that they had good adherence (Supplementary Table S3).

**TABLE 1:** Baseline characteristics of children with rheumatic diseases in groups with good and poor medication adherence.

	Total participants (n = 210)	Good adherence (n = 158)	Poor adherence (n = 52)	*p*-value
Age (years)^a^	14.10 ± 4.74	13.72 ± 4.79	15.25 ± 4.45	0.044^b^
Male sex, n (%)	67 (31.90)	43 (27.22)	24 (46.15)	0.011^b^
Diagnosis				
Connective tissue disease (CTD), n (%)	97 (46.19)	87 (55.06)	10 (19.23)	<0.001^b^
- SLE	77 (36.67)	69 (43.67)	8 (15.38)	<0.001^b^
- JDM	16 (7.62)	16 (10.13)	0 (0)	0.014^b^
- Overlapping syndrome	4 (1.90)	2 (1.27)	2 (3.85)	0.257
Juvenile idiopathic arthritis (JIA), n (%)	111 (52.86)	71 (44.94)	42 (80.77)	<0.001^b^
- SJIA	29 (13.81)	23 (14.56)	6 (11.54)	0.584
- ERA	29 (13.81)	14 (8.86)	15 (28.85)	<0.001^b^
- OligoJIA	22 (10.48)	16 (10.13)	6 (11.54)	0.073
- PolyJIA	33 (15.71)	18 (11.39)	15 (28.85)	0.003^b^
Clinically active disease, n (%)	78 (37.14)	45 (28.48)	33 (63.46)	<0.001^b^
Patients as primary providers, n (%)	103 (49.05)	78 (49.37)	25 (48.08)	0.872
Disease duration (years)^c^	4.33 (2.08–6.98)	3.91 (1.90–6.52)	5.67 (3.31–9.21)	0.005^b^
Types of medication^c^	3 (2–3)	3 (2–3)	3 (2–4)	0.118
Maximum tablets taken per day^c^	4.50 (2.00–7.50)	4.00 (2.00–7.00)	6.00 (3.63–10.38)	<0.001^b^
Subcutaneous medications, n (%)	48 (22.86)	26 (16.46)	22 (42.31)	<0.001^b^
- SC MTX	25 (11.90)	15 (9.49)	10 (19.23)	0.060
- TNF inhibitor	26 (12.38%)	13 (8.23)	13 (25.00)	0.001^b^

a Mean±standard deviation. SLE, systemic lupus erythematosus; JDM, juvenile dermatomyositis; SJIA, systemic JIA; ERA, enthesitis-related arthritis; OligoJIA, oligoarticular JIA; PolyJIA, polyarticular JIA; MTX, methotrexate; TNF, tumor necrosis factor; SC, subcutaneous.

^b^ *p* < 0.05 indicates statistical significance.

^c^ Median (interquartile range).

### 3.3 PRAQ questionnaire

In addition to quantitative methods, we used the PRAQ as a qualitative method to further explore the reasons for medication non-adherence in patients with chronic illness. The PRAQ was administered to both caregivers and patients. For caregivers, PRAQ scores ranged from 0 to 25. Patients in the poor adherence group had significantly higher PRAQ scores than those in the good adherence group (11.00 ± 3.47 vs. 9.51 ± 3.16; *p* = 0.004). We then summed the PRAQ scores in each index and used a cut-off score of 50% to classify patients into groups with high and low index scores for the five indexes of the PRAQ. We compared patients with high index scores between good and poor adherence groups. The results showed that a greater number of patients in the poor adherence group had high index scores in the therapeutic (*p* = 0.002) and relationships (*p* = 0.008) indexes compared to those in the good adherence group (Table 2).

**TABLE 2:** Comparison of Pediatric Rheumatology Adherence Questionnaire (PRAQ) scores and their indexes for caregivers of patients with good and poor adherence.

	Good adherence (n = 158)	Poor adherence (n = 52)	*p*-value
Total scores (25 points)^b^	9.51 ± 3.16	11.00 ± 3.47	0.004^a^
High index score ^c^			
Socioeconomic, n (%)	15 (9.49)	2 (3.85)	0.251
Health system, n (%)	3 (1.90)	0 (0)	1.000
Health conditions, n (%)	92 (58.23)	28 (53.85)	0.580
Therapeutic, n (%)	9 (5.70)	11 (21.15)	0.002^a^
Relationships, n (%)	3 (1.90)	6 (11.54)	0.008^a^

^a^ *p* < 0.05 indicates statistical significance.

^b^ Mean ± standard deviation.

^c^ High index score = total PRAQ, score in each index more than 50%.

According to the Thai version of the PRAQ for pediatric patients with a rheumatic disease, 126 patients who completed the questionnaire had scores ranging from 0 to 20; in total, 73.02% were girls, with a mean age of 17.21 ± 2.62 years and a median disease duration of 6.0 (IQR 3.46–8.92) years (Supplementary Table S4). Approximately half of patients had CTDs and 43.65% had JIA, among which ERA was the most common JIA subtype. One-third of patients had active disease. Patients with good adherence had mean Thai PRAQ scores of 10.86 ± 2.28, whereas patients with poor adherence had mean scores of 12.34 ± 2.89, *p* = 0.003. Comparing patients with high index scores between both groups showed that patients in the poor adherence group had higher scores in the therapeutic index than patients in the good adherence group (*p* = 0.026), as shown in Table 3. The other index scores were not significantly different between the two groups.

**TABLE 3:** Comparison of the Pediatric Rheumatology Adherence Questionnaire (PRAQ) scores and their indexes performed by literate patients with good and poor adherence (n = 126).

	Good adherence (n = 91)	Poor adherence (n = 35)	*p*-value
Total scores (20 points)^b^	10.86 ± 2.28	12.34 ± 2.89	0.003^a^
High index score ^c^			
Socioeconomic, n (%)	15 (16.48)	8 (22.86)	0.407
Health system, n (%)	2 (2.20)	1 (2.86)	1.000
Health conditions, n (%)	84 (92.31)	33 (94.29)	1.000
Therapeutic, n (%)	11 (12.09)	10 (28.57)	0.026^a^
Relationships, n (%)	12 (13.19)	3 (8.57)	0.556

^a^ *p* < 0.05 indicates statistical significance.

^b^ Mean ± standard deviation.

^c^High index score = total PRAQ, score in each index more than 50%.

### 3.4 Barriers to treatment

**FIGURE 2:**
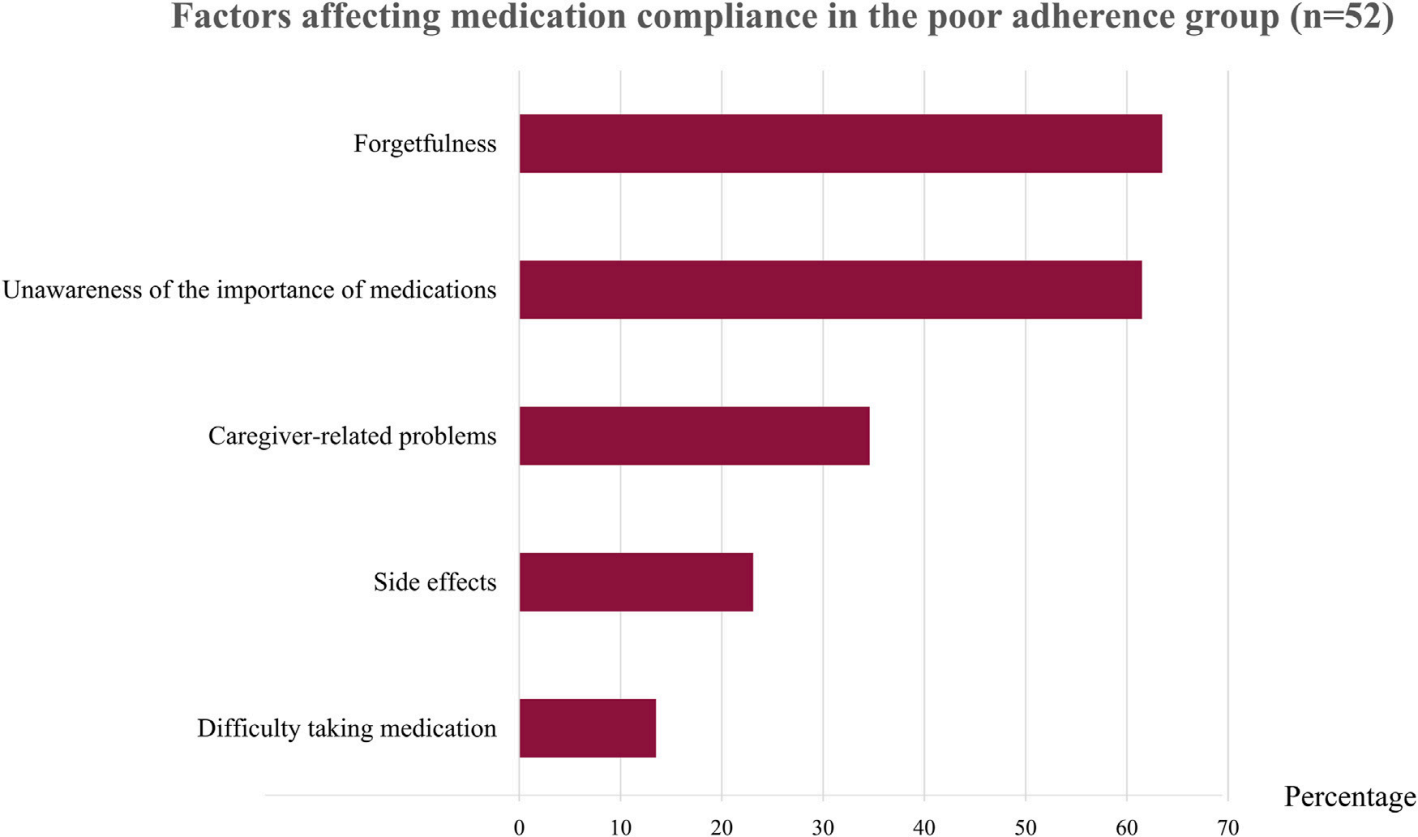
Factors affecting medication compliance in the poor adherence group (n = 52).

After interviewing patients with irregular medication use, we found that the two main reasons for poor adherence were forgetfulness (63.46%) and unawareness about the importance of the medicines (61.54%) (Figure 2 and Supplementary Table S5). Patients who forgot to take their medication explained that they often neglected to take the medicines on time or sometimes forgot to take them to school or to their dormitory. Most patients who thought their medicines were unimportant stated that taking or not taking the medicines did not affect their disease activity. This group of patients included those with inactive ERA who received tumor necrosis factor (TNF) inhibitors and patients with polyarticular JIA and minimal disease activity who received a combination of DMARDs. Another reason for poor adherence in one-third of these patients was that their caregiver did not give them their medicines (34.61%), especially in younger patients. Side effects of the medications (23.08%) was another reason for not taking the drugs, including methotrexate (which causes nausea and vomiting) and prednisolone (which causes weight gain, a moon-face appearance, and hirsutism). Another reason was that patients said the pill shape or method of taking the medication were not compatible with daily activities (13.46%); these included large sulfasalazine tablets, medications to be taken several times a day, and medications to be taken with meals.

### 3.5 Predictors of poor adherence to medical treatment

The factors substantially associated with poor adherence in univariate analysis included ERA, polyarticular JIA, duration of disease ≥5 years, active disease status, forgetfulness, unawareness of the importance of medicines, total PRAQ score for caregivers ≥12, prednisolone, DMARDs, and TNF inhibitors (Table 4). In multivariate analysis, ERA (OR 9.09, 95% CI 1.25–66.18; *p* = 0.029), polyarticular JIA (OR 6.43, 95% CI 1.30–31.75; *p* = 0.022); disease duration ≥5 years (OR 3.88, 95% CI 1.17–12.87; *p* = 0.027), active disease (OR 6.49, 95% CI 1.76–23.99; *p* = 0.005), forgetfulness (OR 14.18, 95% CI 4.21–47.73; *p* < 0.001), unawareness of the importance of medicines (OR 44.18, 95% CI 11.30–172.73; *p* < 0.001), and total PRAQ score for caregivers ≥12 (OR 6.48, 95% CI 1.76–23.82; *p* = 0.005) were predictors of poor adherence. Prednisolone, DMARDs, and TNF inhibitors were not associated with poor adherence in the multivariate analysis (Table 4).

**TABLE 4:** Predictors of poor medication adherence in children with rheumatic diseases.

Factors	Univariate analysis		Multivariate analysis	
	OR (95% CI)	*p*-value	OR (95% CI)	*p*-value
ERA	4.17 (1.85–9.40)	0.001^a^	9.09 (1.25–66.18)	0.029^a^
PolyJIA	3.15 (1.45–6.85)	0.004^a^	6.43 (1.30–31.75)	0.022^a^
Disease duration ≥5 years	2.48 (1.31–4.70)	0.006^a^	3.88 (1.17–12.87)	0.027^a^
Active disease	4.36 (2.25–8.45)	<0.001^a^	6.49 (1.76–23.99)	0.005^a^
Forgetfulness	7.73 (3.86–15.46)	<0.001^a^	14.18 (4.21–47.73)	<0.001^a^
Unawareness of the importance of the medicines	34.51 (13.46–88.49)	<0.001^a^	44.18 (11.30–172.73)	<0.001^a^
PRAQ score for caregivers ≥12	4.30 (2.18–8.49)	<0.001^a^	6.48 (1.76–23.82)	0.005^a^
Prednisolone	0.42 (0.22–0.82)	0.011^a^	1.48 (0.40–5.43)	0.556
Oral MTX	2.62 (1.38–4.97)	0.003^a^	0.68 (0.18–2.54)	0.567
Sulfasalazine	3.00 (1.53–5.91)	0.001^a^	0.61 (0.12–3.16)	0.556
HCQ	0.33 (0.17–0.67)	0.002^a^	0.96 (0.23–4.09)	0.956
TNF inhibitors	3.72 (1.60–8.66)	0.002^a^	1.22 (0.22–6.77)	0.818
Leflunomide	3.92 (1.43–10.79)	0.008^a^	0.28 (0.04–2.11)	0.216

^a^ *p* < 0.05 indicates statistical significance. OR, odds ratio; CI, confidence interval; ERA, enthesitis-related arthritis, PolyJIA, polyarticular juvenile idiopathic arthritis; PRAQ, pediatric rheumatology adherence questionnaire; MTX, methotrexate; HCQ, hydroxychloroquine; TNF, tumor necrosis factor.

## 4 Discussion

Most rheumatic disorders are chronic, and therapy for rheumatic diseases in children is often long-term. Medication non-adherence has been identified as a primary issue among these patients owing to its impact on illness outcomes, quality of life, and cost to the family and healthcare system (McGrady and Hommel, 2013; Len et al., 2014; El-Rachidi et al., 2017). We investigated non-adherence to medical treatment using the pill count method, the PRAQ questionnaire, and interviews to identify relevant variables. One-fourth of patients reported poor medication adherence, which was associated with ERA, polyarticular JIA, active illness, disease duration ≥5 years, and a total PRAQ score ≥12. According to interviews, being unaware of the importance of the medications and forgetting to take them were factors strongly associated with non-adherence to medical treatment.

The frequency of drug non-adherence in rheumatic diseases, especially among children, varies among studies because of different assessment methods with different objectives and benefits. We selected the pill count method and the quantitative measurement because these techniques have been demonstrated to be effective and reliable in prior studies (Pieper et al., 1989; Lam and Fresco, 2015; Driscoll and Modi, 2020; Favier et al., 2020). Self-evaluation of drug use by patients or caregivers was found to be unreliable in this study since around half of patients in the poor adherence group believed that they took more than 80% of their medications as prescribed. The frequency of poor adherence in this study was comparable to rates reported for children with rheumatic diseases in other studies (Pelajo et al., 2012; Nitinantaponk and Oo-puthinan, 2018; Silva et al., 2019). However, some previous studies found higher incidence of poor adherence in caregiver-reported outcomes (Lawson et al., 2011; Bugni et al., 2012) whereas caregivers in this study reported a lower incidence of poor adherence. It is difficult to compare adherence rates among studies using different tools to assess medication adherence. Studies using the Morisky Medication Adherence Scale (MMAS) classified patients as having high, moderate, or low adherence (Senzaki, 2015; Adriano et al., 2017; Keppeke et al., 2018), and studies using other methods classified patients into “good” or “poor” adherence. Moreover, there may be discrepancies among results when using several methods in the same study. Adriano et al. (2017) reported only 4.7% poor adherence using MMAS, but approximately 75% irregular medication use was found in a review of medication dispensing registers in the outpatient pharmacy. Owing to the wide variability of caregiver-reported outcomes, study results should be interpreted with caution and additional quantitative methods should be used to confirm study findings.

The advantage of the PRAQ is that this scale comprises five indexes, corresponding to the five dimensions of the WHO (Sabaté, 2003) regarding medication adherence, to investigate factors associated with poor adherence among children (Silva et al., 2019). This questionnaire was designed specifically for children with rheumatic diseases, and use of this questionnaire in conjunction with objective pill counts improves the effectiveness of assessment and covers all important aspects of drug use. Although the PRAQ has not been validated, the results of this study showed that poor drug adherence was associated with total PRAQ score ≥12. Additionally, the Thai version of the PRAQ applied in the context of children is useful for assessing adolescents who are responsible for taking their own medication.

Caregivers reported that patients with poor adherence also had higher scores in therapeutic and relationship indexes than those with good adherence. The main problem in the therapeutic index was that patients refused to take their medication regularly owing to side effects, having to take many pills, forgetfulness, boredom, and having caregivers that were unable to deal with this issue. Treatment adherence affects family relationships, particularly those involving adolescents. When children have a different perspective on illness and therapy than their parents or when parents have differing opinions, this can also contribute to family conflict (Santer et al., 2014). According to our experience and earlier research (Britton and Moore, 2002; Slatter et al., 2004; Wrubel et al., 2005), a poor relationship between caregivers or parents and their children usually leads to poor adherence. Other factors contributing to poor medication adherence are a rigid regimen, drug management at school, and effects on daily activities (Aston et al., 2019). Our patients also reported difficulties taking drugs in the morning, particularly during the morning rush before school and after breakfast. Some patients were unable to maintain normal activities owing to drug side effects, and others did not want their peers to know about their disease or to feel different. Therefore, individualized treatment plans and a flexible regimen may benefit these individuals (Aston et al., 2019).

ERA and polyarticular JIA were the most common JIA subtypes in this study and both subtypes were associated with poor medication adherence. Around half of patients with ERA who do not respond to standard treatment require biologic agents, including anti-TNF therapy (Vilaiyuk et al., 2016). The indication for anti-TNF therapy in Thailand’s JIA treatment guidelines is failure of two DMARDs within 6 months, which differs from the 2019 ACR treatment guideline for JIA (Ringold et al., 2019) for cost and other economic reasons. Moreover, Thai national health insurance or the UCS do not cover anti-TNF treatment for patients with JIA. Parents must pay for treatment or seek funding for this costly medication. However, patients with ERA receiving anti-TNF treatment did not take the oral medication regularly, particularly sulfasalazine, which comes in the form of a large tablet and patients must take several of these pills daily. Our patients reported that anti-TNF therapy provided a greater response than oral medications. With anti-TNF therapy, the patient’s condition remains inactive, although they do not take oral medications in combination, such as sulfasalazine or methotrexate. Therefore, the most regularly used medication in our study was anti-TNF agents. In contrast to patients with CTDs, chronic arthritis in JIA is less severe than multiple organ involvement in active SLE or JDM. This may better explain the more regular drug use among children with CTDs than in those with JIA.

Disease duration equal to or greater than 5 years and active disease were also associated with poor medication adherence in this study. Longer disease duration leads to patient boredom and lack of interest in disease control, making them less compliant with therapy. Some individuals tire of taking medications because their illness is not cured. Patients with active JIA and a long disease duration, particularly polyarticular JIA, report that taking or not taking their drugs does not make a difference because they still have chronic arthritis regardless of whether they take the medication. If these patients are unable to obtain anti-TNF mediations owing to financial constraints, they may become acclimated to chronic arthritis and adapt their functional abilities to daily routine activities. In a previous study (Sturge et al., 1997), patients with less severe JIA subtypes, such as oligoarticular JIA, exhibited worse medical adherence owing to a lack of motivation to take their medications, in contrast with patients in our study who had more severe JIA subtypes, such as polyarticular JIA. Therefore, the variation in medication adherence among these individuals might differ by region and is influenced by several factors. Long-standing disease duration or late stage of disease are among these factors. Patients with JIA, particularly those with polyarticular JIA, which usually has a long disease duration, have already been treated with NSAIDs and DMARDs and have less pain compared with patients in the early stages of disease, making them less compliant with treatment.

Medication adherence is impacted by the difficulty of the treatment regimen, such as the form of the medication or side effects, or time-consuming or complex treatment regimens, including too many tablets (Bugni et al., 2012; Santer et al., 2014). Although we did not find any specific drugs associated with poor adherence in this study, the most irregularly used drugs in the poor adherence group were sulfasalazine, leflunomide, and mycophenolate mofetil. The latter two are given to patients who fail to respond to conventional treatment. These patients must take many pills per day, which may explain their poor adherence to these additional medications. Thus, the predictors of poor adherence in this study were based on the type and characteristics of rheumatic disease, duration of illness, disease status, and patients’ responsibility more than specific medications.

Various factors contribute to irregular drug use, but forgetfulness and unawareness about the importance of therapy were strongly associated with poor drug adherence. Other studies have found that neglecting to take medicine correctly is the most common barrier to good adherence (Sturge et al., 1997; Lawson et al., 2011; Bugni et al., 2012; Senzaki, 2015; Adriano et al., 2017; Favier et al., 2018). Nevertheless, numerous issues contribute to adherence, including complicated prescriptions (Bugni et al., 2012; Favier et al., 2018), a patient’s lack of concern for their health, caregivers’ perspectives (Adriano et al., 2017; Keppeke et al., 2018; Nitinantaponk and Oo-puthinan, 2018), and a lack of concern about the illness (Santer et al., 2014). Therefore, improving medication adherence requires support for both caregivers and healthcare providers, including a flexible and individualized regimen, strengthening and enhancing family relationships, and providing education about the disease and need for regular treatment, particularly in children with ERA and polyarticular JIA who have the disease for more than 5 years.

Our study had some limitations. First, this study was conducted at a single site, which may not represent all children with rheumatic diseases in Thailand. However, our hospital is the main referral center in Thailand and receives patients from every part of the country. Second, although the PRAQ is a simple tool for assessing drug adherence, it may be prone to recall bias. As a result, we used the pill count method to reduce this bias. Third, because this was a cross-sectional study, a single time point may not represent an individual’s overall adherence; several factors influence and alter medication compliance over time. Therefore, a prospective cohort design and multicenter study are recommended.

## 5 Conclusion

One-fourth of children with rheumatic diseases in our study, particularly those with ERA and polyarticular JIA, tended to have poor medication adherence. Physicians should be aware of suboptimal treatment adherence in patients with long-standing diseases lasting 5 years or more. The use of the PRAQ as a tool for early identification and understanding of the core reasons for noncompliance could be beneficial. Patients who have difficulty following a complex regimen may benefit from a flexible, personalized therapeutic approach. Unawareness about the importance of disease control and consistent treatment, as well as forgetfulness, are critical issues that must be resolved to promote regular drug use. From another perspective, we should not focus only on patients’ culpability for this problem since multiple factors contribute to poor medication adherence, requiring patients, caregivers, and healthcare providers to collaborate and address this issue. In addition, the healthcare system is also the cornerstone in supporting and encouraging patients and their families to enhance treatment compliance (Devine et al., 2018), thereby improving patients’ disease outcomes and quality of life.

## Data Availability

The original contributions presented in the study are included in the article/Supplementary Material, further inquiries can be directed to the corresponding author.

## References

[B1] Adriano L. S. de França Fonteles M. M. de Fátima Menezes Azevedo M. Beserra M. P. P. Romero N. R. (2017). Medication adherence in patients with juvenile idiopathic arthritis. Rev. Bras. Reumatol. Engl. Ed. 57, 23–29. 10.1016/j.rbre.2016.05.004 28137399

[B2] Aringer M. Costenbader K. Daikh D. Brinks R. Mosca M. Ramsey-Goldman R. (2019). 2019 European League against Rheumatism/American College of Rheumatology classification criteria for systemic lupus erythematosus. Arthritis Rheumatol. 71, 1400–1412. 10.1002/art.40930 31385462 PMC6827566

[B3] Aston J. Wilson K. A. Terry D. R. P. (2019). The treatment-related experiences of parents, children and young people with regular prescribed medication. Int. J. Clin. Pharm. 41, 113–121. 10.1007/s11096-018-0756-z 30478490 PMC6394506

[B4] Bohan A. Peter J. B. (1975a). Polymyositis and dermatomyositis (first of two parts). N. Engl. J. Med. 292, 344–347. 10.1056/NEJM197502132920706 1090839

[B5] Bohan A. Peter J. B. (1975b). Polymyositis and dermatomyositis (second of two parts). N. Engl. J. Med. 292, 403–407. 10.1056/NEJM197502202920807 1089199

[B6] Britton C. A. Moore A. (2002). Views from the inside, Part 3: How and why families undertake prescribed exercise and splinting programmes and a new model of the families' experience of living with juvenile arthritis. Br. J. Occup. Ther. 65, 453–460. 10.1177/030802260206501004

[B7] Bugni V. M. Ozaki L. S. Okamoto K. Y. Barbosa C. M. Hilário M. O. Len C. A. (2012). Factors associated with adherence to treatment in children and adolescents with chronic rheumatic diseases. J. Pediatr. (Rio J. 88, 483–488. 10.2223/JPED.2227 23269234

[B8] Devine F. Edwards T. Feldman S. R. (2018). Barriers to treatment: Describing them from a different perspective. Patient Prefer Adherence 12, 129–133. 10.2147/PPA.S147420 29398908 PMC5775743

[B9] Driscoll K. A. Modi A. C. (2020). “Introduction,” in Adherence and self-management in pediatric populations. Editors Modi A. C. Driscoll K. A. (Cambridge, MA: Academic Press), 1–23.

[B10] El-Rachidi S. LaRochelle J. M. Morgan J. A. (2017). Pharmacists and pediatric medication adherence: Bridging the gap. Hosp. Pharm. 52, 124–131. 10.1310/hpj5202-124 28321139 PMC5345910

[B11] Favier L. A. Taylor J. Loiselle Rich K. Jones K. B. Vora S. S. Harris J. G. (2018). Barriers to adherence in juvenile idiopathic arthritis: A multicenter collaborative experience and preliminary results. J. Rheumatol. 45, 690–696. 10.3899/jrheum.171087 29419467 PMC5932234

[B12] Favier L. A. Harry O. Rich K. L. (2020). “Rheumatic diseases,” in Adherence and self-management in pediatric populations. Editors Modi A. C. Driscoll K. A. (Cambridge, MA: Academic Press), 333–353.

[B13] Feldman D. E. De Civita M. Dobkin P. L. Malleson P. N. Meshefedjian G. Duffy C. M. (2007). Effects of adherence to treatment on short-term outcomes in children with juvenile idiopathic arthritis. Arthritis Rheum. 57, 905–912. 10.1002/art.22907 17665485

[B14] Hansen R. A. Kim M. M. Song L. Tu W. Wu J. Murray M. D. (2009). Comparison of methods to assess medication adherence and classify non-adherence. Ann. Pharmacother. 43, 413–422. 10.1345/aph.1L496 19261962

[B15] Keppeke L. F. Molina J. Miotto E Silva V. B. Terreri M. T. S. E. L. R. A. Keppeke G. D. Schoen T. H. (2018). Psychological characteristics of caregivers of pediatric patients with chronic rheumatic disease in relation to treatment adherence. Pediatr. Rheumatol. Online. J. 16, 63. 10.1186/s12969-018-0280-7 30314523 PMC6186042

[B16] Lam W. Y. Fresco P. (2015). Medication adherence measures: An overview. Biomed. Res. Int. 2015, 217047. 10.1155/2015/217047 26539470 PMC4619779

[B17] Lawson E. F. Hersh A. O. Applebaum M. A. Yelin E. H. Okumura M. J. von Scheven E. (2011). Self-management skills in adolescents with chronic rheumatic disease: A cross-sectional survey. Pediatr. Rheumatol. Online. J. 9, 35. 10.1186/1546-0096-9-35 22145642 PMC3254592

[B18] Lazarevic D. Pistorio A. Palmisani E. Miettunen P. Ravelli A. Pilkington C. (2013). The PRINTO criteria for clinically inactive disease in juvenile dermatomyositis. Ann. Rheum. Dis. 72, 686–693. 10.1136/annrheumdis-2012-201483 22736096 PMC5040631

[B19] Len C. A. Miotto E Silva V. B. Terreri M. T. (2014). Importance of adherence in the outcome of juvenile idiopathic arthritis. Curr. Rheumatol. Rep. 16, 410. 10.1007/s11926-014-0410-2 24504596

[B20] Lerkvaleekul B. Apiwattanakul N. Tangnararatchakit K. Jirapattananon N. Srisala S. Vilaiyuk S. (2022). Associations of lymphocyte subpopulations with clinical phenotypes and long-term outcomes in juvenile-onset systemic lupus erythematosus. PLoS. One. 17, e0263536. 10.1371/journal.pone.0263536 35130317 PMC8820627

[B21] McGrady M. E. Hommel K. A. (2013). Medication adherence and health care utilization in pediatric chronic illness: A systematic review. Pediatrics 132, 730–740. 10.1542/peds.2013-1451 23999953 PMC3784296

[B22] Nitinantaponk S. Oo-puthinan S. (2018). Factors associated with adherence to medications for children with juvenile idiopathic arthritis at Queen Sirikit National Institute of Child Health. Thai. J. Pharm. Prac. 10, 100–110.

[B23] Pasma A. Schenk C. Timman R. van 't Spijker A. Appels C. van der Laan W. H. (2017). Does non-adherence to DMARDs influence hospital-related healthcare costs for early arthritis in the first year of treatment? PLoS. One 12, e0171070. 10.1371/journal.pone.0171070 28152001 PMC5289489

[B24] Pelajo C. F. Sgarlat C. M. Lopez-Benitez J. M. Oliveira S. K. F. Rodrigues M. C. F. Sztajnbok F. R. (2012). Adherence to methotrexate in juvenile idiopathic arthritis. Rheumatol. Int. 32, 497–500. 10.1007/s00296-010-1774-x 21246362

[B25] Petty R. E. Southwood T. R. Manners P. Baum J. Glass D. N. Goldenberg J. (2004). International League of associations for rheumatology classification of juvenile idiopathic arthritis: Second revision, edmonton, 2001. J. Rheumatol. 31, 390–392. 14760812

[B26] Pieper K. B. Rapoff M. A. Purviance M. R. Lindsley C. B. (1989). Improving compliance with prednisone therapy in pediatric patients with rheumatic disease. Arthritis Care Res. 2, 132–135. 10.1002/anr.1790020407 2487717

[B27] Ringold S. Angeles-Han S. T. Beukelman T. Lovell D. Cuello C. A. Becker M. L. (2019). 2019 American College of rheumatology/arthritis foundation guideline for the treatment of juvenile idiopathic arthritis: Therapeutic approaches for non-systemic polyarthritis, sacroiliitis, and enthesitis. Arthritis Rheumatol. 71, 846–863. 10.1002/art.40884 31021537 PMC6561114

[B28] Sabaté E. (2003). Adherence to long-term therapies: Evidence for action. Geneva: World Health Organization Press.

[B29] Santer M. Ring N. Yardley L. Geraghty A. W. A. Wyke S. (2014). Treatment non-adherence in pediatric long-term medical conditions: Systematic review and synthesis of qualitative studies of caregivers' views. BMC Pediatr. 14, 63. 10.1186/1471-2431-14-63 24593304 PMC3984727

[B30] Scalzi L. V. Hollenbeak C. S. Mascuilli E. Olsen N. (2018). Improvement of medication adherence in adolescents and young adults with SLE using web-based education with and without a social media intervention, a pilot study. Pediatr. Rheumatol. Online J. 16, 18. 10.1186/s12969-018-0232-2 29540181 PMC5852975

[B31] Senzaki S. V. (2015). Data from: Identifying factors in medication non-adherence in teens diagnosed with juvenile arthritis: A pilot study. SJSU Sch. Digit. Repos. 10.31979/etd.rcju-wdd2

[B32] Silva V. Okamoto K. Y. K. Ozaki L. D. S. Len C. A. Terreri M. (2019). Early detection of poor adherence to treatment of pediatric rheumatic diseases: Pediatric Rheumatology Adherence Questionnaire - a pilot study. Rev. Paul. Pediatr. 37, 149–155. 10.1590/1984-0462/;2019;37;2;00015 30892543 PMC6651309

[B33] Slatter A. Francis S. Smith F. Bush A. (2004). Supporting parents in managing drugs for children with cystic fibrosis. Br. J. Nurs. 13, 1135–1139. 10.12968/bjon.2004.13.19.16318 15573006

[B34] Sturge C. Garralda M. E. Boissin M. Doré C. J. Woo P. (1997). School attendance and juvenile chronic arthritis. Br. J. Rheumatol. 36, 1218–1223. 10.1093/rheumatology/36.11.1218 9402869

[B35] Uribe A. G. Vilá L. M. McGwin G. Jr. Sanchez M. L. Reveille J. D. Alarcón G. S. (2004). The Systemic Lupus Activity Measure-revised, the Mexican Systemic Lupus Erythematosus Disease Activity Index (SLEDAI), and a modified SLEDAI-2K are adequate instruments to measure disease activity in systemic lupus erythematosus. J. Rheumatol. 31, 1934–1940. 15468356

[B36] van Vollenhoven R. Voskuyl A. Bertsias G. Aranow C. Aringer M. Arnaud L. (2017). A framework for remission in SLE: Consensus findings from a large international task force on definitions of remission in SLE (DORIS). Ann. Rheum. Dis. 76, 554–561. 10.1136/annrheumdis-2016-209519 27884822

[B37] Vilaiyuk S. Soponkanaporn S. Jaovisidha S. Benjaponpitak S. Manuyakorn W. (2016). A retrospective study on 158 Thai patients with juvenile idiopathic arthritis followed in a single center over a 15-year period. Int. J. Rheum. Dis. 19, 1342–1350. 10.1111/1756-185X.12637 26176300

[B38] Wallace C. A. Giannini E. H. Huang B. Itert L. Ruperto N. Childhood Arthritis Rheumatology Research Alliance (2011). American College of Rheumatology provisional criteria for defining clinical inactive disease in select categories of juvenile idiopathic arthritis. Arthritis Care Res. Hob. 63, 929–936. 10.1002/acr.20497 21717596

[B39] Wrubel J. Moskowitz J. T. Richards T. A. Prakke H. Acree M. Folkman S. (2005). Pediatric adherence: Perspectives of mothers of children with HIV. Soc. Sci. Med. 61, 2423–2433. 10.1016/j.socscimed.2005.04.034 15936134

