# Supplementary material for: Factors associated with medication adherence among children with rheumatic diseases
Source: Front Pharmacol. 2023 May 5;14:1149320. doi: 10.3389/fphar.2023.1149320 (PMC10196057; doi:10.3389/fphar.2023.1149320)
Supplement: Supplementary file 1 [file DataSheet1.docx]

Supplementary Material

**Factors associated with medication adherence among children with rheumatic diseases**

Roongroj Manatpreeprem, Butsabong Lerkvaleekul, Soamarat Vilaiyuk^*^

* Correspondence: Soamarat Vilaiyuk: soamarat21@hotmail.com

# Supplementary Table

**Table S1** Pediatric Rheumatology Adherence Questionnaire (PRAQ), original and

Thai versions for caregivers and patients.

| **Original version** | **Thai version** | |
| --- | --- | --- |
|  | **Caregiver version** | **Patient version** |
| **Socioeconomic Indexes** | | |
| Q1. Is the distance between your home and the hospital/outpatient service prejudicial to your child's treatment? | Q1. Do you think the distance to the hospital is an obstacle to treating your child? | Q1. Do you think the distance to the hospital causes a problem with your treatment? |
| Q2. Is the means of transportation used to attend visits and exams prejudicial to the treatment? | Q2. Do you think that the transportation used to travel to the hospital (e.g., private car or bus) is an obstacle to the treatment? | Q2. Do you think that the transportation used to travel to the hospital (such as by private car or bus) causes a problem with your treatment? |
| Q3. Is the number of children you have prejudicial to your sick child's treatment? | Q3. Do you think the number of children you have is an obstacle to treating a sick child? | - |
| Q4. Is your work in the home or profession prejudicial to your child's treatment? | Q4. Does working from home or at the office interfere with your child’s treatment? | - |
| Q5. Are visits and exams prejudicial to your work? | Q5. Does a doctor’s visit interfere with your work? | - |
| **Indexes related to the health care team and system** | | |
| Q6. Is it difficult to understand explanations given by the doctor? | Q6. Do you think the doctor’s explanation was hard to understand? | Q3. Did you find it hard to understand what the doctor said? |
| Q7. Does the doctor provide too little information about your child’s condition? | Q7. Do you think the doctor gave too little information about your child’s symptoms? | Q4. Do you think the doctor didn't tell you enough about your condition? |
| Q8. Does the doctor provide too little information on the medication your child is taking? | Q8. Do you think the doctor gave too little information about your child’s medicine? | Q5. Do you think the doctor didn't tell you enough about the medicine you were given? |
| **Indexes related to the health condition** | | |
| Q9. Do you think your child is healthy? | Q9. Do you think your child is healthy? | Q6. Do you think you are healthy? |
| Q10. Do you think your child's complaints are sometimes exaggerated? | Q10. Do you think that sometimes your child exaggerates their symptoms? | - |
| Q11. Do you think that despite their condition, your child has a good quality of life? | Q11. Even if your child is sick because of this disease, do you think your child still has a good quality of life? | Q7. Even if this disease makes you sick, do you think you can still have a good quality of life? |
| Q12. Do you think that despite your child's condition, your family has a good quality of life? | Q12. Even if your child is sick because of this disease, do you think your family still has a good quality of life? | Q8. Even if you get sick from this disease, do you think your family will have a good life? |
| **Indexes related to the therapy** | | |
| Q13. Is your child refusing or has refused any medication? | Q13. Has your child ever refused to take medicine or receive treatment? | Q9. Have you ever refused to take medicine or receive treatment? |
| Q14. Does/did your child have any kind of reaction to a medication? | Q14. Does your child have side effects from taking the medicine or treatment? | Q10. Do the medicines or treatments you're getting make you feel bad? |
| Q15. Is the number of pills your child is taking prejudicial to the treatment? | Q15. Is the quantity or type of medicine an obstacle to the treatment? | Q11. Does the amount or type of medicine you take make it difficult for you to continue your treatment? |
| Q16. Is the type of medication (tablet, solution, intravenous, subcutaneous) prejudicial to your child's treatment? | Q16. Do you think the type of medicine (tablet, syrup, intravenous, subcutaneous injection) is an obstacle to your child’s treatment? | Q12. Do you think the type of medicine you're taking (tablet, syrup, intravenous, or subcutaneous injection) hampers your treatment? |
| Q17. Is the time of the medication prescribed by the doctor a problem for your child's treatment? | Q17. Is the timing of taking the medication provided by the doctor a concern for your child’s treatment? | Q13. Does the time your doctor tells you to take your medicine cause a problem with your treatment? |
| Q18. Do you allow your child to take his/her medication without the assistance of an adult? | Q18. Do you allow your child to take medicine by themself without the supervision of a parent? | Q14. Can you take the medication by yourself without parental help? |
| Q19. Have you forgotten to give your child his/her medication? | Q19. Have you ever forgotten to prepare medicine for your child? | Q15. Have you ever forgotten to take your medicine? |
| Q20. Have you failed to give medication to your child because he/she refused it? | Q20. Have you ever failed to give medicine to your child because your child refused to take it? | - |
| Q21. Have you failed to give medication to your child because he/she was not feeling well? | Q21. Have you ever failed to give medicine to your child because your child felt sick? | Q16. Have you ever missed taking medicine because of feeling sick? |
| Q22. Do you think your child does not need the medication prescribed? | Q22. Do you think your child does not necessarily take the prescribed medication? | Q17. Do you think you don't need the medicine your doctor gave you? |
| Q23. Do you have doubts about the medication your child is taking? | Q23. Do you have concerns regarding the medication that your child is taking? | Q18. Have you ever had questions about the medicine a doctor gave you? |
| **Indexes related to the patient/caregiver** | | |
| Q24. Do you have relationship problems with your child? | Q24. Do you have relationship difficulties with your child? | Q19. Do you have trouble getting along with your parents? |
| Q25. Do you think you are wasting your time with your child's treatment? | Q25. Do you think the treatment of your child is a waste of your time? | Q20. Do you think the treatment is a waste of your time? |

**Table S2** Irregular use of medications by patients in the poor adherence group (n=52).

| Drug name | Number of patients receiving the medications | Number of patients with poor adherence^a^ |
| --- | --- | --- |
| Prednisolone, n (%) | 16 | 12 (75.0) |
| Methotrexate, n (%) | 30 | 21 (70.0) |
| Hydroxychloroquine, n (%) | 13 | 3 (23.08) |
| Azathioprine, n (%) | 8 | 4 (50.0) |
| Sulfasalazine, n (%) | 22 | 22 (100) |
| Leflunomide, n (%) | 9 | 9 (100) |
| Cyclosporine, n (%) | 2 | 0 (0) |
| Mycophenolate mofetil, n (%) | 5 | 5 (100) |
| NSAIDs, n (%) | 27 | 22 (81.48) |
| MTX (subcutaneous), n (%) | 10 | 5 (50.0) |
| TNFi (subcutaneous), n (%) | 13 | 0 (0) |

^a^Poor adherence defined as taking less than 80% of prescribed medications between visits. NSAIDs, non-steroidal anti-inflammatory drugs; MTX, methotrexate; TNFi, tumor necrosis factor inhibitors.

**Table S3** Self-evaluation of medication use by patients in the poor adherence group (n=52).

|  | Good adherence | Poor adherence |
| --- | --- | --- |
| Self/Patients reported, n (%) | 8 (32.0) | 17 (62.96) |
| Caregivers reported, n (%) | 17 (68.0) | 10 (37.04) |
| Total | 25 (48.08) | 27 (51.92) |

Good adherence defined as taking 80% or more of prescribed medication between visits. Poor adherence defined as taking less than 80% of prescribed medications between visits.

**Table S4** Baseline characteristics of literate children completing the Thai children's Pediatric Rheumatology Adherence Questionnaire (PRAQ).

|  | Total participants (n =126) | Good adherence (n=91) | Poor adherence (n=35) | P-value |
| --- | --- | --- | --- | --- |
| Age (years)^‡^ | 17.21 ± 2.62 | 17.02 ± 2.57 | 17.72 ± 2.71 | 0.177 |
| Male, n (%) | 34 (26.98) | 20 (21.98) | 14 (40.0) | 0.041^a^ |
| Diagnosis |  |  |  |  |
| Connective tissue disease (CTDs), n (%) | 71 (56.35) | 63 (69.23) | 8 (22.86) | <0.001^a^ |
| - SLE | 62 (49.21) | 56 (61.54) | 6 (17.14) | < 0.001^a^ |
| - JDM | 6 (4.76) | 6 (6.59) | 0 (0) | 0.185 |
| - Overlapping syndrome | 3 (2.38) | 1 (1.10) | 2 (5.71) | 0.186 |
| Juvenile idiopathic arthritis (JIA), n (%) | 55 (43.65) | 28 (30.77) | 27 (77.14) | < 0.001^a^ |
| - SJIA | 12 (9.52) | 9 (9.89) | 3 (8.57) | 0.561 |
| - ERA | 21 (16.67) | 9 (9.89) | 12 (34.29) | 0.001^a^ |
| - OligoJIA | 8 (6.35) | 5 (5.49) | 3 (8.57) | 0.391 |
| - PolyJIA | 14 (11.11) | 5 (5.49) | 9 (25.71) | 0.003^a^ |
| Active disease, n (%) | 39 (30.95) | 16 (17.58) | 23 (65.71) | <0.001^a^ |
| Patients as primary providers, n (%) | 95 (75.40) | 71 (78.02) | 24 (68.57) | 0.270 |
| Disease duration (years)^¶^ | 6.00 (3.46-8.92) | 5.50 (2.83-7.92) | 7.58 (4.75-10.33) | 0.022^a^ |
| Types of medication^¶^ | 3 (2-3) | 2 (1-3) | 3 (3-4) | 0.002^a^ |
| Maximum tablets taking per day^¶^ | 4.50 (2.00-8.00) | 4.00 (2.00-7.00) | 7.00 (4.00-11.00) | 0.001^a^ |
| Subcutaneous medications, n (%) | 25 (19.84) | 9 (9.89) | 16 (45.71) | < 0.001^a^ |
| - MTX | 10 (7.93) | 4 (4.40) | 6 (17.14) | 0.027^a^ |
| - TNF inhibitor | 16 (12.70) | 5 (5.49) | 11 (31.43) | < 0.001^a^ |

^a^ P<0.05 indicates statistical significance. ^¶^Median (interquartile range). ^‡^Mean±standard deviation. SLE, systemic lupus erythematosus; JDM, juvenile dermatomyositis; SJIA, systemic JIA; ERA, enthesitis-related arthritis; OligoJIA, Oligoarticular JIA; PolyJIA, Polyarticular JIA; MTX, methotrexate; TNF, tumor necrosis factor.

**Table S5** Factors affecting medication compliance in the poor adherence group (n=52).

| **Reasons** | **Number of patients, n (%)** |
| --- | --- |
| Forgetfulness | 33 (63.46) |
| Unawareness of the importance of the medications | 32 (61.54) |
| Caregiver related problem | 18 (34.61) |
| Side effects | 12 (23.08) |
| The difficulty of taking medication (pill shape, method) | 7 (13.46) |

**Note: Additional explanations from the interviews.**

**Forgetfulness**

- I did not take drugs with my breakfast because I got up late.
- I forgot to bring medication to school to take during the day.
- I was busy with something else, so I did not take my drugs on time.

**Unawareness of the importance of medications**

- Because I had been on the drugs for a long time, I got bored. I stopped taking drugs for a few days, and my symptoms were still the same, so I stopped taking them.
- I thought I was healthy and did not need medications.
- I thought anti-tumor necrosis factor agents worked better than oral medications, so I stopped taking them, especially sulfasalazine.

**Caregiver-related problems**

- If my caregiver forgot to give me the medicine, I did not take it.
- I saw my child get sick after taking methotrexate, so I didn't give it to him/her.

**Side effects**

- I got sick every time I took methotrexate, so I only took it sometimes.
- I could not stand the smell and taste of methotrexate. Every time I see the medication, I feel sick and have nausea.
- I did not like taking steroids because they made me gain weight and increased my appetite.
- I did not want to take methotrexate because I felt sick and could not do daily activities.

**Difficulty taking medication**

- I found it hard to swallow a big pill (sulfasalazine, mycophenolate mofetil), so I only took it sometimes.
- There were too many pills, so I only took some of them.
- I forgot to take the medicine before a meal, so I skipped that dose.
